# Cytokine-driven glycosphingolipid metabolism modulates endoplasmic reticulum calcium homeostasis in primary human renal mesangial cells

**DOI:** 10.3389/fimmu.2025.1688916

**Published:** 2025-12-10

**Authors:** Mariia Stefanenko, Tessa M. Ortiz, Sandra G. Mungaray, Mykhailo Fedoriuk, Oleg Palygin, Stefano Berto, Drew Moore, Tamara K. Nowling

**Affiliations:** 1 Division of Rheumatology, Department of Medicine, Medical University of South Carolina, Charleston, SC, United States; 2 Division of Nephrology, Department of Medicine, Medical University of South Carolina, Charleston, SC, United States; 3 Department of Neuroscience, Medical University of South Carolina, Charleston, SC, United States

**Keywords:** mesangial cell, endoplasmic reticulum stress, glycosphingolipid, cytokines, lupus

## Abstract

**Introduction:**

Glycosphingolipids (GSLs), including hexosylceramides (HexCers), lactosylceramides (LacCers), and gangliosides composed of one or more sugar residues attached to ceramide, are essential components of cell membranes. Dysregulated GSL metabolism has been implicated in various inflammatory and autoimmune diseases, including lupus nephritis; however, its contribution to renal cell dysfunction remains largely unexplored.

**Methods:**

Primary human renal mesangial cells (hRMCs) were treated with proinflammatory cytokines IL-1β, TNFα, IFNγ, and/or IFNα in the absence or presence of eliglustat, an FDA-approved pharmacological inhibitor of GSL synthesis. Effects on HexCers levels, cell viability, and cytokine secretion were evaluated by high-performance liquid chromatography-tandem mass spectrometry, alamar blue, and ELISAs respectively. Gene expression was determined by bulk RNA sequencing. Cytosolic and endoplasmic reticulum (ER) Ca^2+^ levels were measured by Fluo-8 fluorescent dye and laser scanning confocal microscopy.

**Results:**

Stimulation of hRMCs with proinflammatory cytokines relevant to lupus elicited significant upregulation and secretion of inflammatory mediators that parallel intracellular and extracellular accumulation of HexCers and elevated cytosolic calcium (Ca^2+^) levels. The increase in cytosolic Ca^2+^ was attributed to a decrease in endoplasmic reticulum (ER) Ca^2+^ store capacity. Pharmacological inhibition of GSL synthesis with eliglustat significantly reduced HexCers levels and restored ER Ca^2+^ stores, but did not impact cytokine-induced cytokine/chemokine secretion or cell viability/proliferation.

**Conclusion:**

Together, these data suggest that elevated GSL synthesis modulates cytokine-induced ER Ca^2+^ dysregulation in mesangial cells and may play a role in the pathogenesis of lupus nephritis.

## 1 Introduction

Systemic lupus erythematosus (SLE) is an autoimmune disease that causes heterogeneous patterns of inflammation in the human body through generation of autoantibodies and deposition of immune complexes. Development of renal disease or lupus nephritis (LN) leads to increased proteinuria, renal fibrosis, and eventual organ failure. LN occurs in approximately half of patients diagnosed with SLE (1), and only 50% of LN patients respond well to standard therapies (2–4). Glomeruli are the initial site of immune complex deposition in LN.

Glomeruli are key structures of the kidney and the initial site of immune complex deposition in LN (1). The glomerular filter is composed of fenestrated capillary endothelium, glomerular basement membrane, and podocyte foot processes. Mesangial cells (MCs) comprise approximately one-third of cells in the glomerulus, providing structural support through the production of matrix proteins, regulating glomerular blood flow and filtration rate, and serving as resident immune-like cells by performing functions such as phagocytosis and production of cytokines (5, 6). In addition to their function as early responders to inflammation, MCs can influence the response and function of other glomerular cells including podocytes by releasing cytokines under normal and pathological conditions (6–9). MC production of cytokines and chemokines promotes immune cell recruitment, eventually leading to mesangial expansion/sclerosis and interstitial fibrosis (10). Despite the key roles that MCs play in glomerular function, the precise cellular and molecular mechanisms that underlie MC response to inflammatory insult are not well understood, especially in LN.

With respect to lupus nephritis, MCs were shown to respond to anti-dsDNA antibodies, lupus serum, or various lupus-relevant cytokines/chemokines with increased proliferation and mesangial matrix production, secretion of proinflammatory cytokines/chemokines and fibrotic factors, or upregulation of an endoplasmic reticulum response (ER) stress pathway (11–15). Glycosphingolipid (GSL) synthesis begins in the ER where the hexosylceramides GlcCers and galactosylceramides are generated by the addition of glucose or galactose, respectively, to ceramide (16). Overproduction and accumulation of ceramide can induce ER stress and disrupt Ca^2+^ homeostasis (17, 18). Levels of GSLs are elevated in the urine and kidney of human patients with LN (13, 19–21) suggesting GSL metabolism plays a role in LN. Most GSLs are located in lipid rafts within the plasma membrane of all cell types and play important roles in intracellular signaling (17); however, their role in modulating MC function is not fully understood. We previously demonstrated that human MCs with higher levels of GSLs had an enhanced response to sera from patients with LN including significantly elevated intracellular Ca^2+^ flux (21).

In this report, we further investigated the role of GSL metabolism in MC response to inflammatory stimuli. We demonstrate that the GSLs hexosylceramides and lactosylceramide are significantly increased in primary human mesangial cells in response to cytokine stimulation that parallels secretion of a large number of cytokines and chemokines, and an increase in cytosolic calcium (Ca^2+^) levels due to reduced ER Ca^2+^ store capacity. Inhibiting GSL metabolism prevented the cytokine-induced reduction of ER Ca^2+^, but did not have an effect on cytokine secretion, cell viability/proliferation or gene expression. These results suggest that MCs respond to inflammatory stimuli by increasing GSL metabolism to modulate ER stress response at a nongenomic/post-transcriptional level.

## 2 Methods

### 2.1 Reagents and kits

Purchased reagents and kits include: recombinant, animal-free human IL-1β (#AF-200-01B), IFNγ (#AF-300-02), IFNα (#01-672-628), and TNFα (#AF-300-01A) (Fisher Scientific, Waltham, MA), Alamar blue (#A50101 Invitrogen, Waltham, MA), eliglustat hemitartrate (#50-187–3104 Fisher Scientific/MedChemexpress), and anti-αSMA monoclonal antibody (17H19L35, #701457 Invitrogen); Alexa Fluor Plus 488-conjugated goat anti-rabbit IgG (H+L), highly cross-adsorbed secondary antibody (#A32731 Invitrogen); Fluoromount-G with DAPI (#00-4959-52, Invitrogen) (Fisher Scientific); and Rhodamine Phalloidin (#PHDR1, Cytoskeleton, Inc., Denver, CO). Human cytokine ELISA kits, IL-6 (#430516), CCL2/MCP-1 (#438804), CXCL8/IL-8 (#431504), CXCL5 (#440904) were purchased from BioLegend (San Diego, CA). RNAeasy kit and QiaShredders were purchased from Qiagen (Santa Clarita, CA).

### 2.2 Cell culture and treatments

Primary human renal mesangial cells (hRMCs) were purchased from ScienCell Research Laboratories (#4200, Carlsbad, CA); lot 17544, fetal female; lot 12445, fetal male; lot 20018, adult male; lot 20057, adult male. Because hRMCs derived from kidneys of adult females were not available commercially, adult female hRMCs (HK31 and HK32) were established as follows. Glomeruli were isolated using a sieving technique (22) by the MUSC Kidney Translational Research Center, which receives discarded transplant kidneys in a de-identified manner. The center meets the Not Human Research criteria and is therefore, not subject to oversight as determined by the MUSC Institutional Review Board. Glomeruli were then cultured to establish hRMC lines, as previously described (23) (**Supplementary Figure S1**). Cells were frozen down in MCM media +10% DMSO at passages 2–3 and stored in the vapor phase of liquid nitrogen. Vials were thawed and placed into culture and passaged using the same protocol as for the hRMCs purchased from ScienCell. At passage 5, cells were characterized visually for morphology and phenotypically using cell-specific markers, and compared to the hRMCs purchased from ScienCell (**Supplementary Figure S1**). Cells were fixed with 4% paraformaldehyde in PBS (Alfa Aesar, #J61899) for 10 minutes at room temperature. Permeabilization was performed using 0.5% Triton X-100 (Bio-Rad, #1610407) in PBS containing 2% BSA for 5 minutes at room temperature. Following permeabilization, cells were blocked with 2% BSA in PBS. Primary antibody staining was performed using anti-α-Smooth Muscle Actin (α-SMA) recombinant rabbit monoclonal antibody at a dilution of 1:100 overnight at 4 °C. After, cells were incubated with Alexa Fluor Plus 488-conjugated goat anti-rabbit IgG (H+L) at 1:500 for 1 hour at room temperature. Afterward, cells were incubated with Rhodamine Phalloidin (100 nM working solution). Each step was followed by washing with PBS. Finally, cells were mounted using Fluoromount-G with DAPI.

All hRMC lots were grown and passaged in low glucose mesangial cell medium (MCM; #4201 ScienCell) supplemented with cell growth supplement MsCGS, penicillin/streptomycin, and 2% FBS according to manufacturer’s recommendations in humidified 5% CO_2_, 37 °C. For experiments, hRMCs at passages 5–7 were plated on 96-well or 6-well plates as indicated in figure legends and grown until ~80% confluent. Treatments were done in triplicate within each experiment (unless otherwise noted) and results from replicate wells averaged. Cells were then refed with fresh medium containing IFNγ (100 ng/ml), IFNα (100 ng/ml), TNFα (100 ng/ml), or IL-1β (25 ng/ml) or a mix of all four cytokines (“cytokine mix”) at the indicated concentrations unless otherwise noted. Eliglustat at the concentrations indicated in the figures/figure legends was added at the same time as the cytokines. Cells were incubated with cytokines +eliglustat or vehicles for 24-72h as indicated in the figures, media was collected, and cell viability measured using the alamar blue assay as described below. Media was used to measure levels of secreted cytokines and normalized to relative cell viability within an experiment or used to measure excreted hexosylceramides (HexCers) or lactosylceramides (LacCers). Cells were collected and RNA isolated for sequencing or used to measure HexCers or LacCers.

### 2.3 Cytokine/chemokine array and ELISAs

Media from two independent experiments, one performed with hRMC lot 17544 and one performed with hRMC lot 20057 treated with cytokines, were used to screen a cytokine/chemokine protein array. hRMCs within each experiment were treated in triplicate on a 96-well plate with vehicle, individual cytokines, or cytokine mix (as described above). Media was collected and pooled from the triplicate wells, the pooled media from each experiment (n=2) was sent to Eve Technologies (Calgary, Canada), and the Human Cytokine/Chemokine 71-plex Discovery Assay Array (HD71) was screened. Cell viability was measured using the alamar blue assay after media was collected. Quantifications provided by Eve Technologies were normalized to cell viability. Individual ELISAs for IL-6, MCP-1 (CCL2), IL-8 (CXCL8), or CXCL5 were performed according to manufacturer’s instructions using media collected from the individual triplicate wells. Final concentrations calculated from the ELISAs were normalized to cell viability and results from triplicate wells averaged.

### 2.4 Cell viability/proliferation

The alamar blue assay was performed at the end of each experiment and used to normalize for cell number differences in ELISAs and lipid measures, and to assess effects of treatments on cell proliferation and survival. In brief, media was collected at the indicated endpoint of each experiment and replaced with fresh medium containing 10% alamar blue and incubated. Fluorescence was measured over time according to the manufacturer’s instructions and background levels in media only (no cells) were subtracted. Fluorescence measurements were used to calculate relative cell viability setting the vehicle-treated (unstimulated) cells to 1 and all other wells compared to the vehicle-treated wells within an experiment. Manual counting of cells confirmed relative numbers as determined by alamar blue in vehicle-treated compared to cells treated with cytokines and/or eliglustat.

### 2.5 Lipidomics

Quantification of hexosylceramides (HexCers) and lactosylceramides (LacCers) was performed on cells and media as previously reported (13, 19, 21) by the Medical University of South Carolina Lipidomics Shared Resource core facility. Quantitative analyses were based on eight-point calibration curves generated for each target analyte (24). Briefly, cells or media was spiked with synthetic standards and extracted. A set of internal standards were spiked into an artificial matrix, subjected to an identical extraction procedure as the biological samples. All samples were then analyzed by high-performance liquid chromatography-tandem mass spectrometry (HPLC/MS/MS) operating in a multiple reaction-monitoring (MRM) positive ionization mode. The analyte/internal standard peak area ratios were plotted against analyte concentrations to generate analyte specific calibration curves. Any lipid with no authentic standards was quantitated using the calibration curve of its closest counterpart. HexCers and LacCers levels in media alone (not incubated with cells), which was nearly undetectable, was subtracted from levels in media incubated with cells. Lipid levels in cells and media were normalized to cell viability measured by the alamar blue assay or to cell number determined by manual counting as indicated in figure legends.

### 2.6 RNA sequencing and data analyses

hRMCs (lots 12445, 17544, and 20018) were treated in quadruplicate wells in 6-well plates with vehicle, 25 ng/ml IL-1β, or 25 ng/ml IL-1β +20 nM eliglustat for 24, 48, or 72 hrs. Media was collected for ELISAs and GSL quantification, cell viability was measured by the alamar blue assay, and cells scraped into DPBS and replicate wells combined. The cell suspension was split into two tubes, centrifuged, and DPBS removed. Half the cells were used for GSL quantification, and the other half were used for RNA isolation. RNA was isolated using RNeasy kit with QiaShredders, resuspended in RNase free water and the concentration and quality assessed by Nanodrop. A total of 2 μg RNA per sample was shipped to Novogene for library preparation and sequencing after passing their quality control (QC) checks. Analyses of the sequencing results were performed by the Bioinformatics Core Facility at the Medical University of South Carolina.

RNA-seq reads were aligned to the human reference genome (hg38) using STAR (v2.7.1a) (25) For each sample, a BAM file was generated containing both mapped and unmapped reads across splice junctions. Secondary alignments and multi-mapped reads were removed using in-house scripts, and only uniquely mapped reads were retained for downstream analyses. GENCODE annotation for hg38 (version 32) was used for reference-guided alignment and downstream quantification. Gene-level expression was quantified using *featureCounts* (v2.0.1) (26), based on protein-coding genes from the annotation files. Quality control metrics were assessed MultiQC (v1.0.dev0) (27). For differential expression, counts were normalized using counts per million reads (CPM). Genes without reads in either sample were removed. The variance explained by biological covariates, such as age and sex, were removed using a linear regression. Variance partition for each gene was calculated using the R package *variancePartition* (28). Differential expression analysis was performed in R using a linear model followed by a *post-hoc* multiple comparison using the R package *emmeans*. Model comprise P-values were adjusted for multiple comparisons using the Benjamini-Hochberg correction (FDR<0.05). Analysis was performed for each time point (24hr, 48hr, 72hr). Genes were considered differentially expressed by FDR < 0.05 and |log_2_ Fold Change| > 0.3 to capture potentially meaningful transcriptional alterations that are characteristic of cell systems, where even modest expression changes can have functional significance due to gene regulatory network and dosage/treatment effects. This threshold has also been used in several recent bulk and single-cell transcriptomic studies (29–32). Downstream analyses, including gene differential expression and gene ontology enrichment, were performed using FDR-corrected p-values, which prioritize statistical robustness. However, we verified that applying a more stringent cutoff (|log_2_FC| > 0.5) yielded highly consistent biological findings, supporting the robustness of our findings (data not shown).

To identify modules of co-expressed genes in the RNA-seq data, we carried out weighted gene co-expression network analysis (WGCNA) (33). A signed network was constructed, and a soft-thresholding power was automatically calculated to achieve approximate scale-free topology (R2 > 0.85). Networks were constructed using the blockwiseModules function with biweight midcorrelation (bicor). Modules were subsequently determined using the dynamic tree-cutting algorithm. A minimum module size of 50 was chosen to allow for the detection of smaller modules, which might otherwise be obscured or deemed noise in the adjusted data. A deep split parameter of 4 was employed to facilitate a more aggressive partitioning of the data, thereby creating more specific modules. Spearman’s rank correlation was used to compute module eigengene–covariate associations. Modules were visualized based on the rank of the topological overlap weight. For visualization purposes, the top 100 connections were selected. Node size in the visualizations was adjusted based on the degree (i.e., number of links). For GO and enrichment analyses, functional annotation of differentially expressed and co-expressed genes was performed using *scToppR* (v0.99.1) (34). Benjamini-Hochberg FDR (FDR<0.05) was applied as a multiple comparison adjustment. Functional categories were filtered based on FDR < 0.05 threshold.

### 2.7 Calcium (Ca^2+^) measures

For Ca^2+^ imaging experiments, hRMCs were cultured on glass-bottom dishes (MatTek, No. 0 coverslip). Cells were pretreated for 24 hours with a cytokine mixture consisting of TNFα, IFNα, and IFNγ (100 ng/ml each), and IL-1β (25 ng/ml) or IL-1β alone (25 ng/ml) with or without the addition of eliglustat (1 nM) for 24 hrs. Following treatment, cells were loaded with Fluo-8 fluorescent dye (#21090, AAT Bioquest) and incubated at 37°C for 1 hour. The medium was then replaced with a Ca^2+^-free extracellular solution (145 mM NaCl, 4.5 mM KCl, 2 mM MgCl_2_, 10 mM HEPES; pH 7.35). To assess ER Ca^2+^ store capacity, 500 nM Thapsigargin (TG; Invitrogen, #T7459), a classical SERCA inhibitor, was acutely applied. Imaging was performed using a Leica TCS SP5 laser scanning confocal microscope equipped with an HCX PL APO CS 40×/NA 1.25 oil immersion objective. Changes in fluorescence intensity were quantified by measuring the mean gray value in individual cells over time. Regions of interest (ROIs) were defined for individual cells across all imaging fields, and analysis was performed using ImageJ (Fiji package). Data were summarized using OriginPro 2021b software (OriginLab, Northampton, MA, USA).

### 2.8 Statistical analyses

Comparison of differences in secreted cytokine levels, proliferation/cell viability, or cell and excreted GSLs levels were performed using GraphPad PRISM 10 (GraphPad Software, Inc., La Jolla, CA). Non-parametric one-way or two-way ANOVA or individual t-tests were performed with adjustment for multiple comparisons as appropriate. All analyses were unpaired. Specific analyses used and P values are noted in the figure legends or figures. Bioinformatics statistics (Benjamini-Hochberg, Spearman’s rank correlation, and hypergeometric test) and programs were performed using R as detailed in the corresponding sections above.

## 3 Results

### 3.1 Cytokine and chemokine secretion by hRMCs in response to interferon (IFN), TNFα, and IL-1β

**Figure 1:**
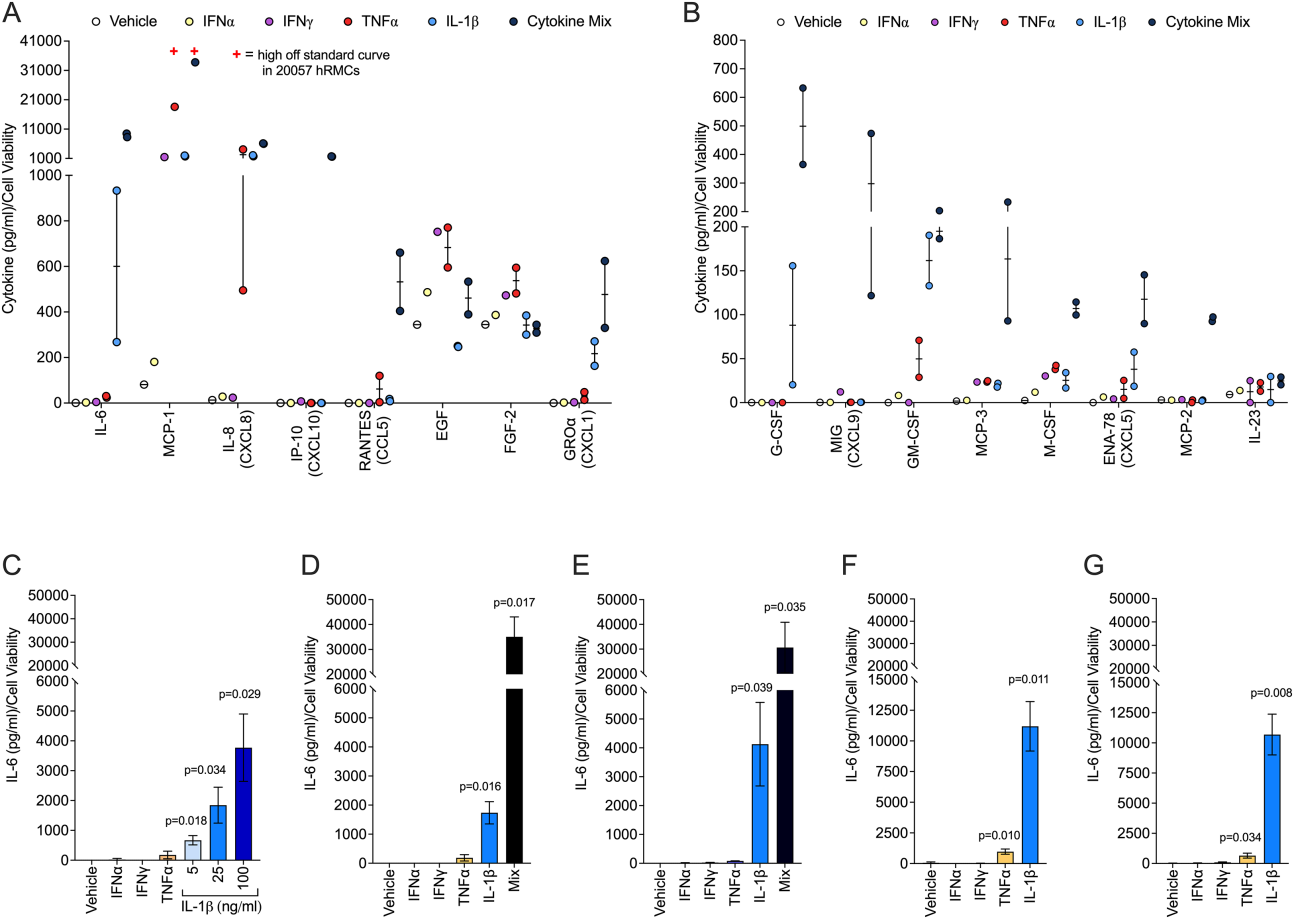
hRMCs secrete many cytokines/chemokines in response to proinflammatory cytokine stimuli. Triplicate wells were treated with IFNα (100 ng/ml), IFNγ (100 ng/ml), TNFα (100 ng/ml), IL-1β, (25 ng/ml), mix of all 4 cytokines (cytokine mix), or vehicle (dH2O) as indicated on the graphs. The media was collected 24h post-stimulation and cell viability measured by the alamar blue assay. For **(A, B)**, media was screened using a 71-plex cytokine/chemokine array. Each point represents an independent experiment, one with lot 17544 and one with lot 20057. Cytokine concentrations were normalized to cell viability and are presented relative to vehicle-treated samples. The 8 most highly expressed factors **(A)** and next 8 most highly expressed factors **(B)** based on expression levels in response to the cytokine mix are presented. “+” = high off the standard curve for one of the experiments. For **(C–G)**, IL-6 levels measured by ELISA in 5 independent experiments each using a different lot of cells: **(C)** lot 20018; **(D)** lot 20057; **(E)** lot 17544; **(F)** lot HK31; **(G)** lot HK32. Concentrations were normalized to cell viability. Media from each triplicate well was assayed independently and the mean +SD is presented. P-values were determined by unpaired T-test between vehicle and each stimulant.

Previously, we demonstrated that hRMCs respond to sera from LN patients with active disease by secreting a variety of cytokines (21). To better evaluate specific mechanisms that underlie cellular response to inflammatory stimuli, we treated two independent lots of commercially available hRMCs with 100 ng/ml each of IFNα, IFNγ, and TNFα, or 25 ng/ml of IL-1β, or a mix of all four cytokines. These four cytokines are produced by activated immune cells or resident renal cells and upregulated in the circulation or kidneys of patients with LN (35–38). Thus, these cytokines were used to model a lupus inflammatory environment *in vitro*. We screened a 71-plex array of cytokines and chemokines with media from two different hRMC lots cultured with each cytokine or the mix of four cytokines for 24hrs. The cytokine predictably elicited significant secretion of a large number of cytokines/chemokines followed by IL-1β and TNFα. The 16 most highly secreted cytokines and chemokines are shown in **Figures 1A, B**. Individual ELISAs for IL-6 (**Figures 1C–G**), MCP-1 and IL-8 (see section 3.5), and CXCL5 (data not shown) confirmed their upregulation in multiple hRMC lines in subsequent experiments. **Figure 1C** shows that the hRMCs respond in a dose-dependent manner to IL-1β in secretion of IL-6, and that only 25 ng/ml IL-1β is required to elicit a significant response while the other cytokines elicited little to no IL-6 even at 100 ng/ml. The cytokine mix elicited a synergistic effect over the individual cytokines (**Figures 1D, E**). Based on these results, we used IL-1β at 25 ng/ml or the cytokine mix (25 ng/ml IL-1β and 100 ng/ml of IFNγ, IFNα, and TNFα) as the inflammatory stimulus for subsequent experiments.

### 3.2 IL-1β treatment significantly increases cellular and excreted levels of HexCers that are reduced by treatment with eliglustat

**Figure 2:**
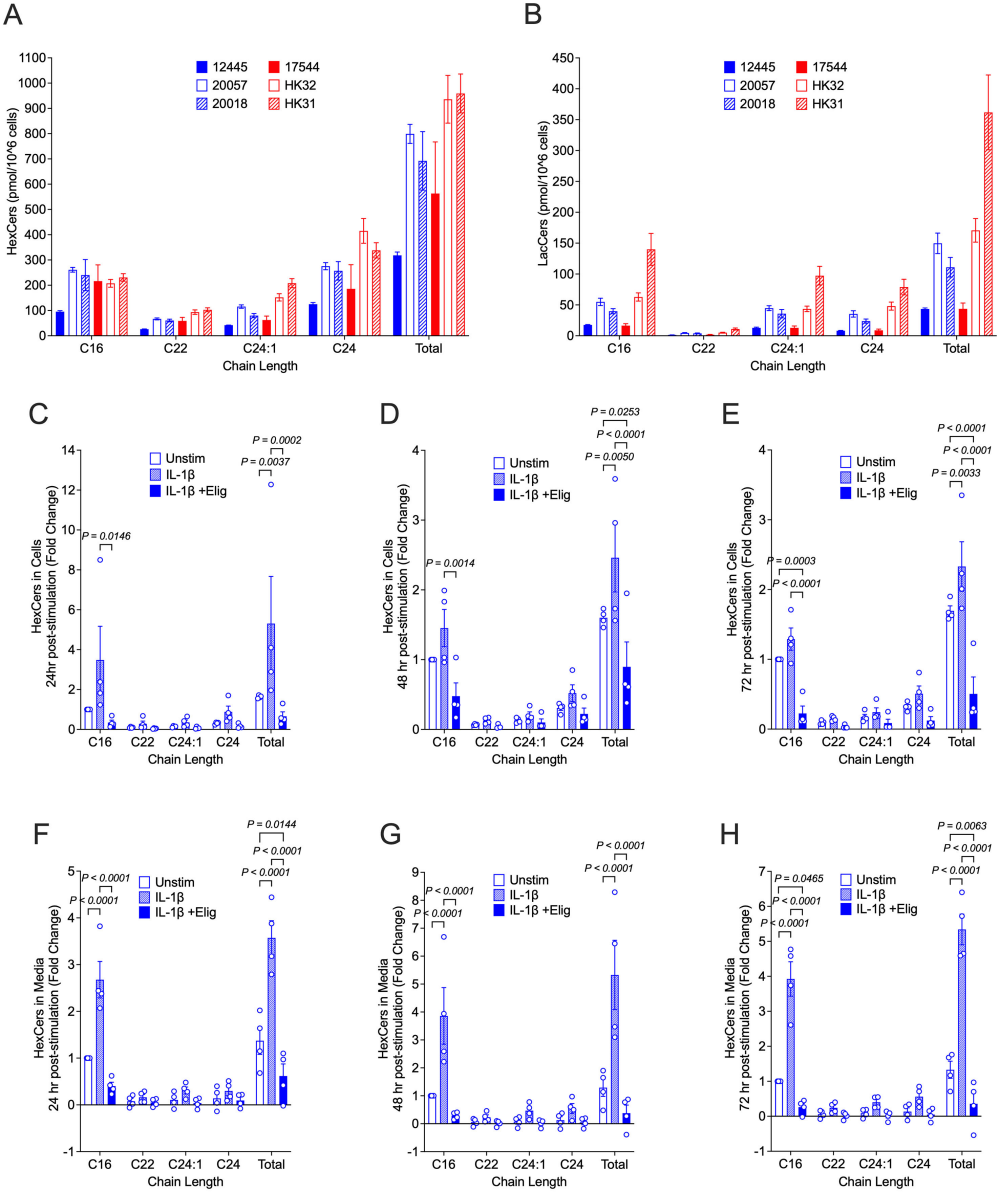
HexCers levels increase in response to IL-1β and reduced by eliglustat in hRMCs. For **(A, B)**, all 6 lots of cells were grown to ~90% confluency in duplicate, trypsinized, counted, and HexCers **(A)** and LacCers **(B)** measured in 1x10^6 cells by high-performance liquid chromatography-tandem mass spectrometry (HPLC/MS/MS). Major chain lengths expressed in the cells are presented. Bars are mean +SD of duplicate wells. For **(C–H)**, hRMCs lots 17544, 12445, 20057, and 20018 were treated in quadruplicate with vehicles, IL-1β, or IL-1β +20 nM eliglustat for 24h **(C, F)**, 48h **(D, G)**, or 72h **(E, H)**. Media was collected, cell viability measured by the alamar blue assay, and cells collected. Quadruplicates within each experiment were pooled and HexCers in cells **(C–E)** and media **(F–H)** measured as in **(A, B)**. HexCers measured in the culture media alone (was not incubated with cells) was subtracted from the media levels in **(F–H)**. Levels were then normalized to cell viability and fold change calculated setting vehicle-treated to 1 for each experiment (hRMC lot). Fold change for the three lots were averaged and mean +SD presented. P-values were determined by two-way ANOVA with Tukey’s multiple comparisons test.

We previously observed that response to inflammatory stimuli in the fetal female hRMCs was more robust than in fetal male hRMCs, which had lower levels of HexCers (21). Thus, we measured cellular levels of HexCers and LacCers in unstimulated hRMCs obtained from 4 adult individuals (2 male, 2 female) compared to the fetal female and fetal male that we analyzed previously. For a more direct comparison, all lots were at passage 6, collected at ~90% confluence, with GSLs measured in the same analysis run, and levels normalized to cell number. This data shows that in unstimulated cells, GSLs vary across lots (individuals) (**Figures 2A, B**), and the variability is more pronounced for LacCers than for HexCers. Since HexCers (GalCers + GlcCers) are the initial GSLs generated from ceramide, we assessed changes to HexCers levels in stimulated hRMCs. Following stimulation with IL-1β, cellular HexCers were significantly increased 24h (**Figure 2C**), 48h (**Figure 2D**), and 72h (**Figure 2E**) post-stimulation with similar fold changes observed across all hRMC lots. Additionally, significant increases were observed in excreted HexCers levels (**Figures 2F–H**). These results suggest IL-1β stimulation induces GSL synthesis and that cellular excretion of excess GSLs into the media may serve to try and maintain homeostatic cellular levels.

Eliglustat, an inhibitor of glucosylceramide (GlcCer) synthase that catalyzes the generation of GlcCer from ceramide, dose-dependently reduced HexCers levels in hRMCS (**Supplementary Figure S2A**). Blocking GSL synthesis with 20 nM eliglustat significantly reduced IL-1β-stimulated increases in cellular (**Figures 2C–E**) and excreted (**Figures 2F–H**) HexCers levels at all time points post-stimulation. This indicates that IL-1β-stimulated increases in HexCers is due largely to increases in GlcCer (rather than GalCer) and supports our previous observations that elevated HexCers in the kidneys of lupus-prone mice are due to increased GlcCers (19). Neither eliglustat alone (**Supplementary Figure S2B**) nor IL-1β alone or in combination with eliglustat (**Supplementary Figure S2C**) had significant effects on proliferation/cell viability.

### 3.3 IL-1β upregulates expression of genes involved in endoplasmic reticulum stress response, but eliglustat has no effect on gene expression

To investigate the transcriptional effects of IL-1β stimulation and its modulation by eliglustat, we performed time-course RNA-seq analyses and systems-level network modeling. To achieve this, RNA was isolated from unstimulated (vehicle-treated), IL-1β-stimulated, and eliglustat-treated IL-1β-stimulated hRMCs at 24-, 48-, and 72-hours post-treatment from three different hRMC replicates. Principal component analysis (PCA) demonstrated remarkable similarity among these replicates, irrespective of treatment or time point (**Supplementary Figure S3A**). Moreover, most of the variance between vehicle- and IL-1β-treated cells was attributable to treatment rather than to the sex or age of the individual from whom the hRMCs were derived (**Supplementary Figure S3B**). These analyses also indicated that the IL-1β-induced gene expression changes were consistently maintained over the 24–72-hour period.

**Figure 3:**
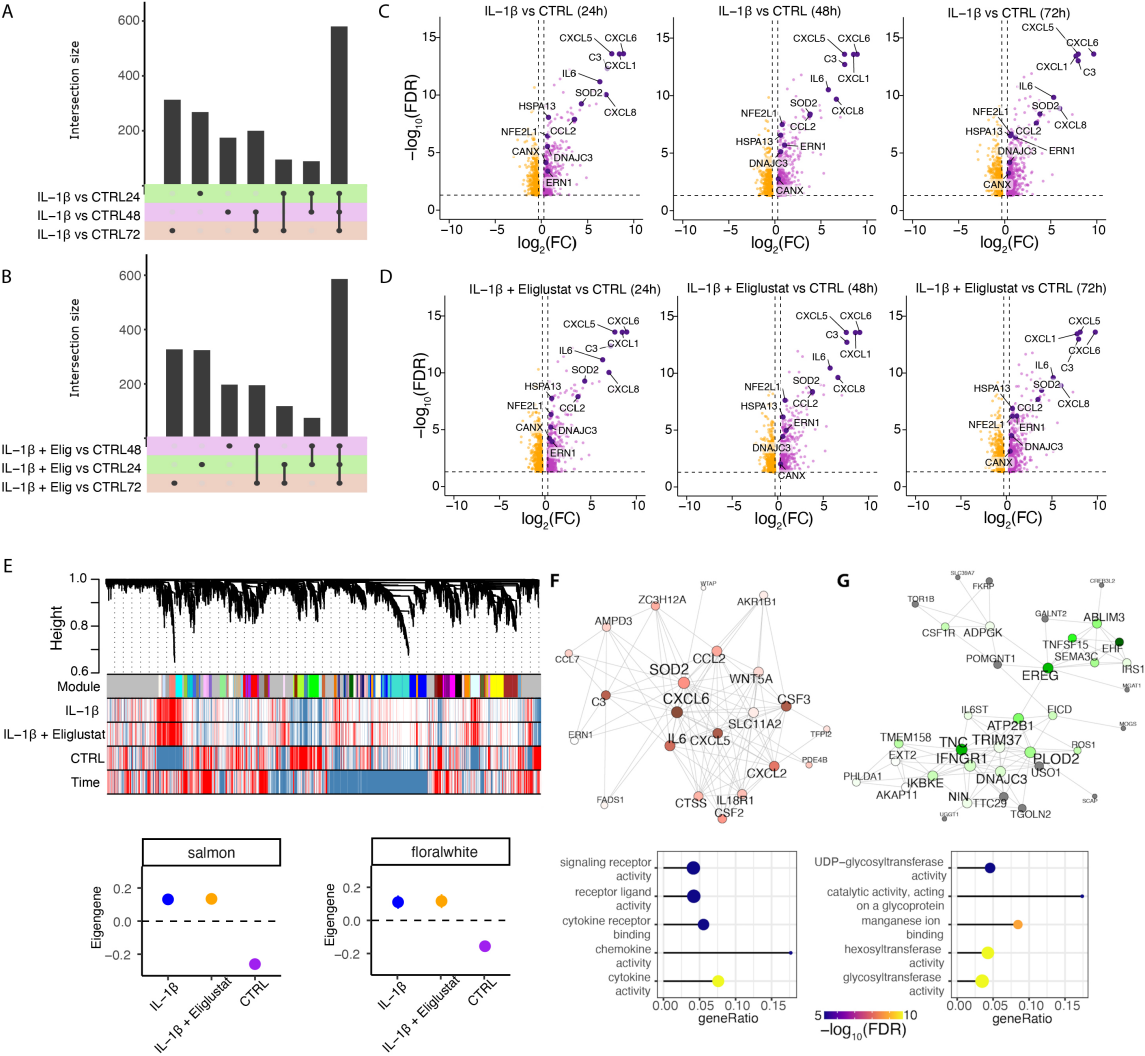
IL-1β upregulates expression of genes in ER stress pathways in hRMCs. hRMCs lots 20018, 12445, and 17544 were treated with vehicle, 25 ng/ml IL-1β, or 25 ng/ml IL-1β +20 nM eliglustat for 24h, 48h, or 72h in quadruplicate and RNA sequencing performed. **(A–B)** UpSet plots showing shared and unique differentially expressed genes between IL-1β and untreated **(A)**, and IL-1β +eliglustat and untreated **(B)** samples across different time points. **(C, D)** Volcano plots displaying significantly upregulated (purple) and downregulated (orange) genes following IL-1β treatment **(C)** and IL-1β +eliglustat treatment **(D)**. Highlighted genes are associated with immune response and ER functions. **(E)** Representative network dendrogram showing correlation patterns between detected co-expression modules, treatments, and time points, and below, dot plots with standard errors (SEs) illustrating the association of selected modules. SEs are calculated based on the module eigengenes across samples. Dots represent the mean eigengene expression for each module. **(F, G)** Network visualization of the top 200 connections ranked by weighted topological overlap for the salmon **(F)** and floralwhite **(G)** modules. Node size corresponds to the number of edges (degree). Enriched pathways for biological process are shown below each module. CTRL= vehicle-treated.

Differential gene expression analysis revealed that IL-1β robustly induced pro-inflammatory transcriptional programs compared to vehicle-treated cells, with the highest number of differentially expressed genes (DEGs) detected at 24 hours and sustained through 72 hours (**Figure 3A**). Co-treatment with IL-1β and eliglustat also resulted in a markedly altered DEG profile compared to vehicle-treated cells over time (**Figure 3B**). Upregulated (purple) or downregulated (orange) genes following IL-1β treatment at 24-, 48-, and 72-hours compared to vehicle-treated cells are shown in **Figure 3C** (FDR <0.05 and |log2(fold change)| >0.3). Interestingly, the addition of eliglustat to IL-1β-stimulated cells did not substantially alter the gene expression signatures induced by IL-1β alone, including those of enzymes involved in glycosphingolipid (GSL) synthesis and key inflammatory mediators such as CXCL1, CXCL5, CXCL6, CCL2, and IL-6 (**Figure 3D**). This suggests that altering GSL levels using eliglustat does not impact IL-1β-induced transcriptome changes in MCs.

We next examined gene expression differences at the systems level using Weighted Gene Co-expression Network Analysis (WGCNA). This approach identified gene modules associated with specific treatment conditions. In total, 25 distinct co-expression modules were detected (**Supplementary Figure S3C**), each representing clusters of genes with coordinated expression patterns across time and treatment groups (**Figure 3E**). Among these, the salmon (**Figure 3F**) and floralwhite (**Figure 3G**) co-expression modules showed the strongest association with IL-1β stimulation, with eigengene expression markedly increased in IL-1β-treated samples compared to vehicle-treated controls.

Network analysis was used to examine the biological significance of the salmon and floralwhite modules. The salmon module (**Figure 3F**) was enriched for canonical pro-inflammatory pathways, including cytokine-mediated signaling and chemokine activity, with central nodes such as CXCL6, IL-6, and SOD2 forming highly interconnected hubs. These genes are known mediators of innate immune activation and endoplasmic reticulum (ER)-related processes, reinforcing the inflammatory nature of this module and its responsiveness to IL-1β. The floralwhite module (**Figure 3G**) showed enrichment for genes in ER-related processes such as glycoprotein metabolism and glycosylation. For example, UDP-glycosyltransferases are involved in glycosylation of ceramide and control of ER-associated degradation (ERAD) (39, 40). The distinct enrichment profiles of these modules highlight the nature of the IL-1β transcriptional response, driving both inflammatory signaling and metabolic remodeling, and importantly, that effects of reducing glycosphingolipids by eliglustat are not due to changes in gene expression.

### 3.4 Blocking GSL synthesis restores cytokine-induced reduction of ER Ca^2+^ stores

**Figure 4:**
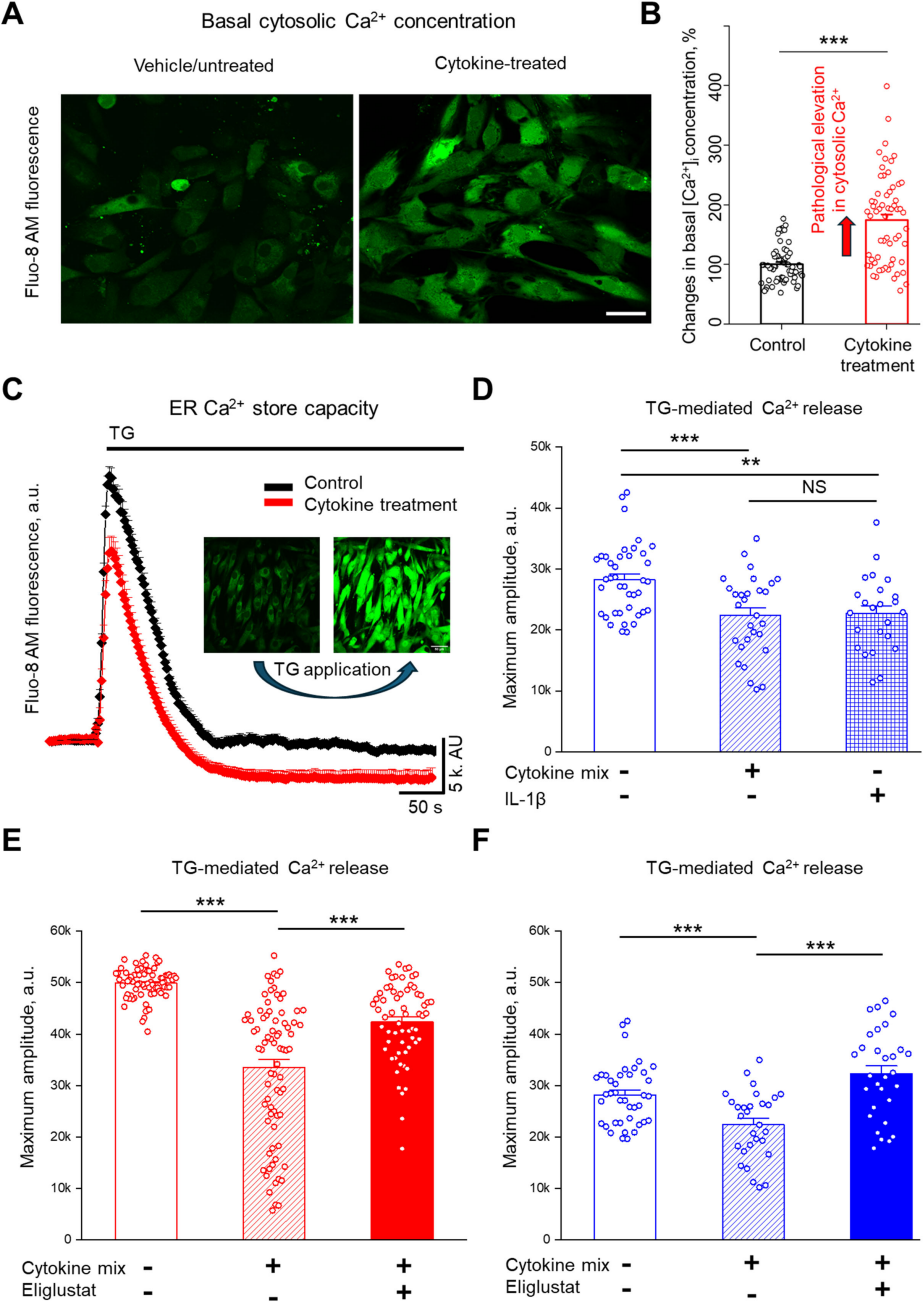
Cytokine treatment increases cytosolic Ca^2+^ and reduces ER Ca^2+^ store capacity, and inhibiting GSL synthesis restores ER Ca^2+^ stores in primary hRMCs. **(A)** Representative images of hRMC lot 20018 loaded with Fluo-8 AM under control conditions (top) and after cytokine treatment (bottom) with the cytokine mix of TNFα, IFNα, IFNγ, and IL-1β for 24 h. Scale bar = 50 µm. **(B)** Quantification of basal cytosolic Ca^2+^ levels, shown as percent of mean control values. Data represent mean +SEM of 4 independent experiments (n ≥ 57 cells); ***p< 0.001 by unpaired t-test. **(C)** Representative Ca^2+^ response trace from Fluo-8 AM–loaded male hRMCs (n ≥ 10 cells) under control (black) and cytokine-treated (red) conditions using Thapsigargin (TG; 500 nM) to assess ER Ca^2+^ store capacity. **(D)** Quantification of TG-induced Ca^2+^ release in hRMCs treated with vehicle (control), IL-1β alone, or the cytokine mix for 24 h. Both IL-1β and cytokine mix significantly reduced ER Ca^2+^ release amplitude compared to vehicle. Bars represent mean +SEM (N = 3 independent experiments; n ≥ 30 cells). Changes in Ca^2+^ amplitude in response to TG were measured in **(E)** HK31 hRMCs and **(F)** 20018 hRMCs after treatment with cytokine mix with or without eliglustat (1 nM) for 24 hr. Bars represent mean +SEM of 3 independent experiments (n ≥ 30 cells per group). ***p< 0.001, **p< 0.01 vs. control by one-way ANOVA with Tukey’s *post hoc* test. ns, not significant.

ER is the major site of Ca^2+^ storage and regulates Ca^2+^ homeostasis in the cell and the cellular stress response (41). We demonstrated previously that hRMCs with elevated levels of GSLs were more responsive to sera from patients with LN that included a significant increase in intracellular Ca^2+^ flux (21). To evaluate the effects of pro-inflammatory cytokines on intracellular Ca^2+^ homeostasis, hRMCs were treated for 24 hours with IL-1β or the cytokine mix. Representative confocal images (**Figure 4A**) revealed an increase in basal cytosolic Ca^2+^ levels following cytokine treatment, which was further confirmed by quantitative analysis (**Figure 4B**). Basal cytosolic Ca^2+^ levels, presented as percent of mean control values, were significantly elevated in cytokine-treated cells compared to untreated controls (***p < 0.001).

To determine the effects of cytokine exposure on ER Ca^2+^ store capacity, Thapsigargin (TG; 500 nM), a specific inhibitor of the sarco/endoplasmic reticulum Ca^2+^-ATPase (SERCA) pump, was applied acutely. By inhibiting SERCA, TG blocks Ca^2+^ reuptake into the ER, leading to a passive leak of stored Ca^2+^ into the cytosol (42). Quantification of the maximum Ca^2+^ amplitude revealed that both IL-1β and the full cytokine mix significantly reduced ER Ca^2+^ stores compared to vehicle-treated controls (**Figures 4C, D**; **p < 0.01 for IL-1β; ***p < 0.001 for cytokine mix vs. control). The difference between IL-1β and the cytokine mix was not statistically significant (NS).

To assess whether GSL synthesis contributes to cytokine-induced depletion of ER Ca^2+^ stores, hRMCs were co-treated with eliglustat for 24 hours. In hRMCs derived from a female donor (HK31; **Figure 4E**) and a male donor (20018; **Figure 4F**), cytokine mix exposure significantly reduced ER Ca^2+^ store capacity compared to untreated controls. Eliglustat-cytokine co-treatment significantly reversed the cytokine effect and increased the magnitude of TG-evoked ER Ca^2+^ release in both cell lines compared to cytokine-treatment only (**p < 0.01, ***p < 0.001). These results suggest that cytokine-mediated disruption of ER Ca^2+^ stores in hRMCs involves GSL-dependent mechanisms, and that pharmacologic inhibition of GlcCer synthase may modulate ER stress under inflammatory conditions. Moreover, since eliglustat does not alter IL-1β-induced changes in the transcriptome, the eliglustat-mediated reversal of IL-1β-induced reduction of ER Ca^2+^ likely involves post-transcriptional or lipidomic mechanisms.

### 3.5 Blocking GSL synthesis does not impact cytokine secretion

**Figure 5:**
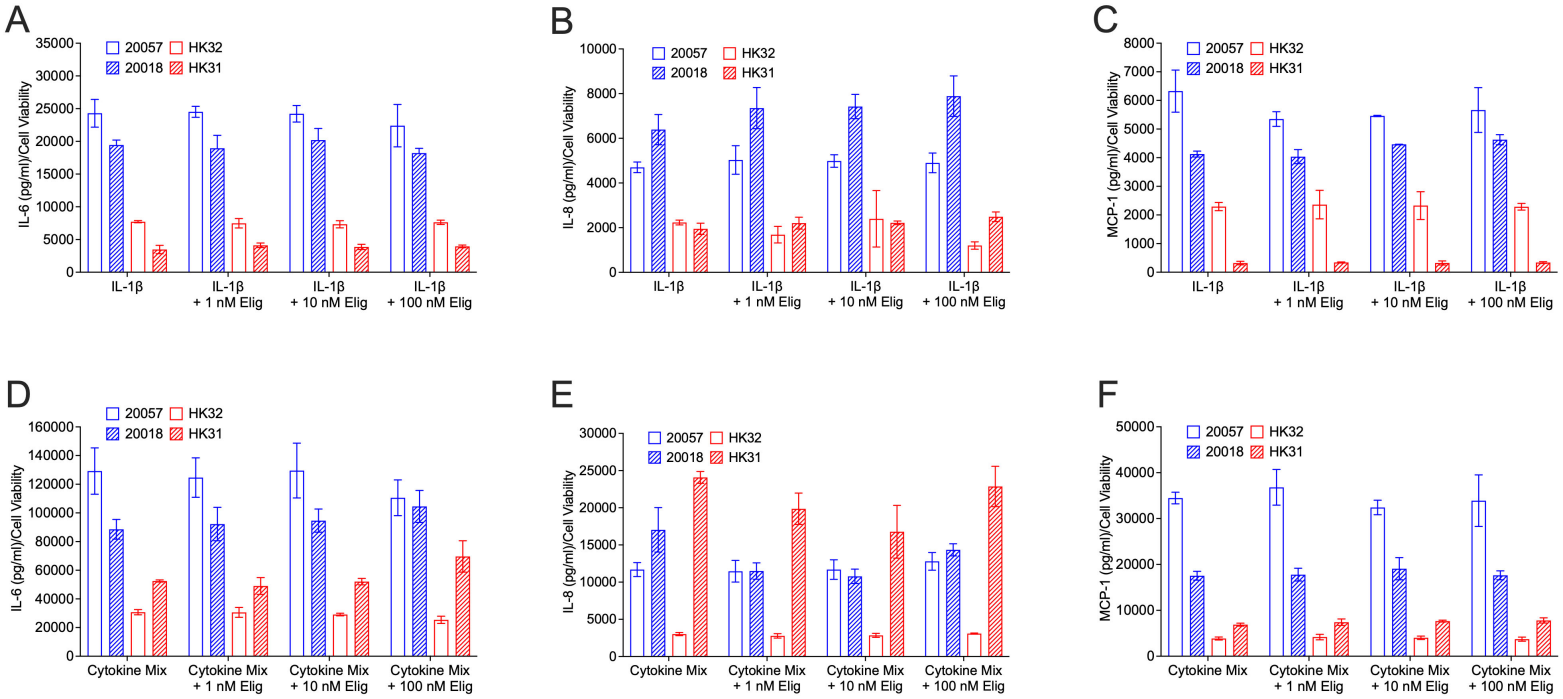
Eliglustat does not significantly impact IL-6, IL-8, MCP-1 release in response to IL-1β or cytokine mix at 24 hours post-treatment. hRMC lots 20057, 20018, HK31, and HK32 were treated with: **(A–C)** IL-1β, IL-1β plus 1 nM, 10 nM, or 100 nM eliglustat, or vehicles in triplicate; or **(D–F)** cytokine mix, cytokine mix plus 1 nM, 10 nM, or 100 nM eliglustat, or vehicles in triplicate. Media was collected after 24h, cell viability measured by alamar blue, and IL-6 **(A, D)**, IL-8 **(B, E)**, or MCP-1 **(C, F)** measured in the media by ELISA. Data is the mean of the triplicate wells +SD.

Although we did not observe effects of eliglustat on IL-1β-induced cytokine expression at the transcriptional level, to determine if blocking GSL production impacts cytokines post-transcriptionally, levels of secreted cytokines were measured. hRMCs were treated with IL-1β (**Figures 5A–C**) or cytokine mix (**Figures 5D–F**) in the absence or presence of increasing concentrations of eliglustat. IL-6 (**Figures 5A, D**), IL-8 (**Figures 5B, E**), and MCP-1 (**Figures 5C, F**), which were significantly increased both transcriptionally (**Figure 3**) and post-transcriptionally (secreted, **Figure 1**) in response to IL-1β, were measured in the media 24 hrs post-stimulation. Despite the significant impact of eliglustat on ER Ca^2+^ store capacity, secretion of these highly expressed cytokines in response to IL-1β or cytokine mix was not significantly impacted. These results suggest that GSL synthesis modulation of the cytokine-induced ER stress response is separate from the expression and release of proinflammatory cytokines under inflammatory conditions.

## 4 Discussion

**Figure 6:**
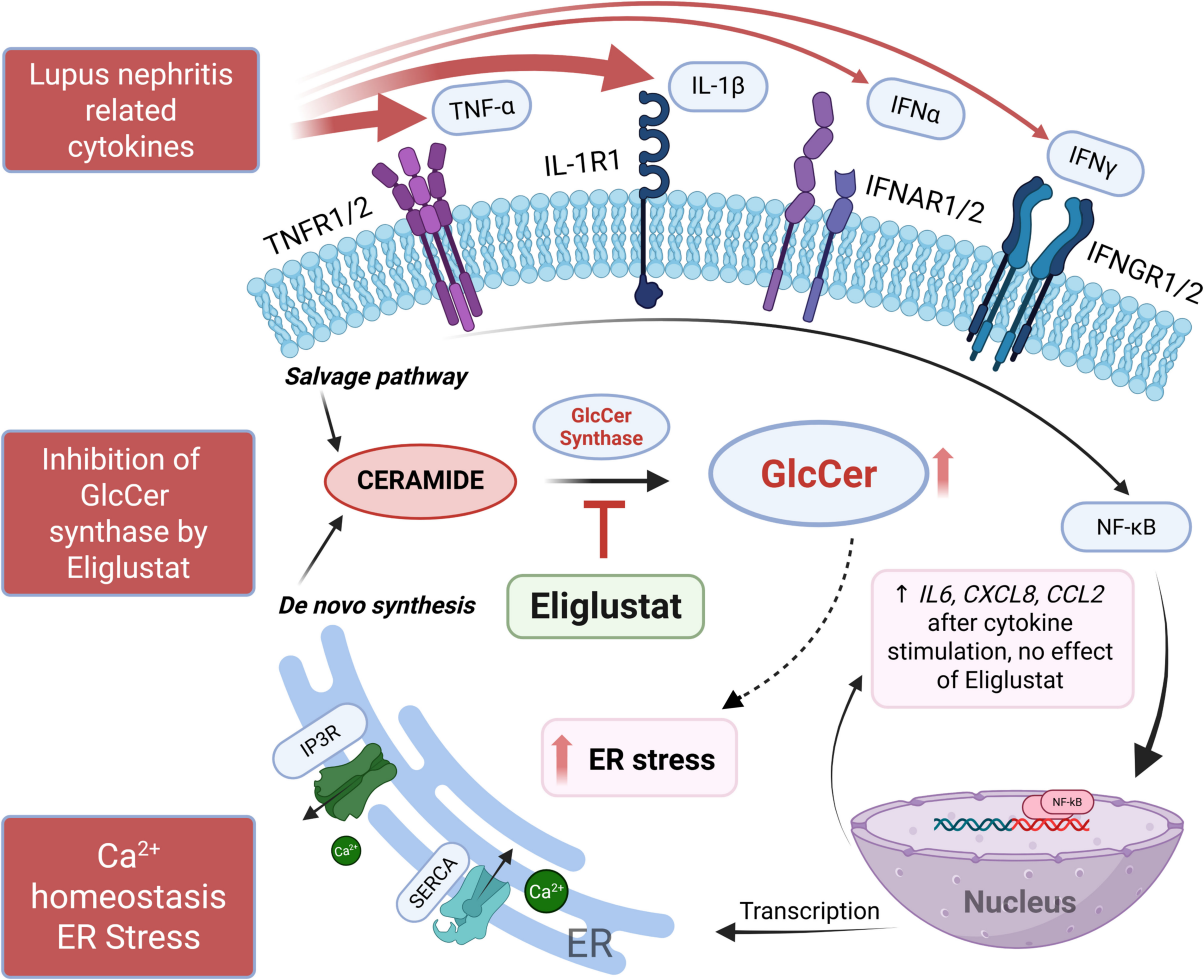
Summary schematic of results. IL-1β and TNFα, and a mix of cytokines (IL-1β, TNFα, IFNα, and IFNγ) elicited the highest response from hRMCs as measured by secretion of multiple cytokines. IL-1β or the mix of cytokines increased GSLs HexCers and LacCers, upregulated proinflammatory pathways and genes/pathways associated with ER-related functions, increased cytosolic Ca^2+^, and decreased ER Ca^2+^ capacity. Eliglustat, an inhibitor of GlcCer synthase that catalyzes the generation of GlcCer from ceramide, reduced HexCers and LacCers and restored ER Ca^2+^ capacity, but did not have an effect on cytokine secretion in response to IL-1β or the mix of cytokines. Thus, eliglustat likely inhibits or reverses cytokine-induced ER stress in hRMCs. Figure created using BioRender.com.

GSLs are elevated in several kidney diseases including LN, and targeting GSL metabolism to reduce GSL levels was shown to improve disease manifestations in several mouse disease models (13, 19, 21, 43–45). While aberrant ER stress signaling was shown to disrupt immune homeostasis in autoimmunity including lupus (15), few studies focused on the ER stress response mechanisms in renal cells (11, 46, 47). Moreover, the role of GSLs with respect to ER stress remains incompletely understood, especially in renal glomerular cells. Thus, our novel study provides significant insight by demonstrating that GSL metabolism plays a role in modulating the ER stress response in hRMCs that can be reversed by inhibiting the GSL synthesis pathway without altering the transcriptome or cytokine production. Our results are summarized in **Figure 6**.

Stress response in cells can manifest in multiple ways, including disruption of ER function that includes regulating Ca^2+^ homeostasis. The ER serves as a Ca^2+^ reservoir, releasing Ca^2+^ through several receptors/channels when concentrations are low in the cytosol and transporting it from the cytosol into the ER through the sarcoplasmic-ER Ca^2+^ ATPase (SERCA) pump when cytosolic levels are elevated (48). Altered Ca^2+^ homeostasis in response to stressors such as ischemia, infection, or toxins can result in accumulation of misfolded or unfolded proteins that quickly accumulate contributing to multiple diseases including autoimmunity, cancer, and kidney diseases (49–51). Our findings demonstrate that pro-inflammatory cytokine exposure leads to disturbed ER Ca^2+^ handling in hRMCs, as evidenced by increased basal cytosolic Ca^2+^ and reduced TG-induced Ca^2+^ release. These alterations reflect ER Ca^2+^ depletion, a pathological condition implicated in multiple diseases due to its impact on protein folding, ER stress responses, and activation of cell death pathways. ER Ca^2+^ depletion was shown to trigger compensatory mechanisms involving store-operated Ca^2+^ entry (SOCE), oxidative stress, and disruption of mitochondrial function (52). In the context of kidney inflammation, impaired ER Ca^2+^ homeostasis may contribute to mesangial dysfunction, altered cytokine secretion, and progression of glomerular injury.

In addition to disrupting Ca^2+^ dynamics, ER stress initiated by cytokine-mediated Ca^2+^ depletion may further exacerbate inflammatory responses. The unfolded protein response (UPR), triggered by ER stress, plays a pivotal role in modulating cytokine production and shaping immune signaling pathways. Activation of UPR sensors such as IRE1α, PERK, and ATF6 can directly influence transcriptional and post-transcriptional regulation of pro-inflammatory mediators, including IL-6, TNFα, and IFNβ (53). ER stress characterized by increased expression of eIF2α and ATF4, molecules in the PERK pathway, was observed in MCs in response to polymeric IgA with respect to renal inflammation in IgA nephropathy (54). In LN, anti-dsDNA antibodies were shown to significantly increase expression of PERK, ATF4, and eIF2α (11). Similarly, the RNAseq data presented above shows upregulation of ERN1, the gene that encodes IRE1α in another pathway of the UPR. In MCs, this may result in a feed-forward loop where ER stress not only reflects cellular dysfunction but actively contributes to the propagation of inflammation and tissue injury. These mechanisms highlight the potential for ER Ca^2+^ homeostasis to act as both a target and regulator of inflammatory signaling in glomerular disease contexts.

The finding that GSL inhibition produced a pronounced effect on Ca^2+^ flux in hRMCs exposed to pro-inflammatory cytokines highlights the importance of inflammatory context in shaping cellular responses to metabolic perturbation. Cytokine treatment may sensitize Ca^2+^-handling machinery by initiating subthreshold activation of the unfolded protein response (UPR), thereby lowering the threshold for stress-induced signaling cascades. Prior studies have shown that inflammatory cytokines can induce moderate UPR activation without triggering apoptosis, through upregulation of sensors such as PERK and IRE1α (53, 55). In this primed state, MCs may become more vulnerable to disruptions in membrane lipid composition, including altered GSL availability, which could further destabilize ER Ca^2+^ homeostasis and exacerbate cellular dysfunction.

The lack of effect of eliglustat on cytokine secretion despite its strong impact on ER Ca^2+^ store capacity suggests that GSL-dependent regulation of ER stress signaling and Ca^2+^ homeostasis may be mechanistically distinct from pathways controlling cytokine synthesis and release. It also suggests that GSLs may primarily modulate intracellular signaling processes associated with protein folding, ER function, and Ca^2+^ storage rather than directly influencing NF-κB or MAPK-dependent transcriptional programs driving cytokine production (as suggested by our transcriptomics data). From a pathophysiological perspective, this finding highlights that GSL remodeling could contribute to mesangial cell dysfunction and stress adaptation under inflammatory conditions without directly altering cytokine-mediated production. A role for GSL metabolism in modulating ER stress in resident renal cells with respect to inflammatory kidney disease has not been previously demonstrated.

Beyond their role in plasma membrane architecture, GSLs contribute to the regulation of inter-organelle communication via mitochondria-associated membranes (MAMs), which serve as functional hubs for Ca^2+^ transfer between the ER and mitochondria (56). Disruption of GSL biosynthesis may destabilize these contact sites, impairing Ca^2+^ flux and promoting mitochondrial depolarization, oxidative stress, and cell injury. Given the dependence of mesangial cells on coordinated ER-mitochondrial signaling for matrix remodeling and survival, perturbation of GSL-regulated MAM integrity may represent a novel mechanism contributing to glomerular injury and remains to be explored.

GSL species in addition to GlcCer and LacCer, including gangliosides GD3 and GM2, are reduced following eliglustat exposure (57, 58). While the specific impact on hRMC membrane architecture remains to be elucidated, these observations support a broader role for GSL biosynthesis in regulating Ca^2+^-related signaling pathways. Gangliosides, a subclass of GSLs, differentially regulate ER stress signaling through distinct effects on Ca^2+^ homeostasis and membrane organization. GM1 has been implicated in the modulation of ER-mitochondrial Ca^2+^ dynamics, where its accumulation can promote mitochondrial depolarization and apoptotic signaling under stress conditions (56). In contrast, excessive GM2 was shown to disrupt ER Ca^2+^ uptake by inhibiting SERCA activity, contributing to ER Ca^2+^ depletion and maladaptive UPR activation (59). These ganglioside-specific effects underscore the importance of lipid composition in shaping ER stress responses and may represent an underexplored layer of regulation during inflammatory or metabolic challenges. Altered ganglioside profiles, particularly in cytokine-exposed mesangial cells, could therefore amplify ER dysfunction and sensitize cells to injury. RNAseq results from our study identified a manganese ion binding co-expression module strongly associated with IL-1β stimulation. Both GM1 synthase and GM2 synthase were shown to require divalent cation manganese for maximum functionality (60).

The glomerulus is comprised of endothelial cells and podocytes in addition to MCs in close proximity to each other and cell-cell communication likely plays an important role in cellular response. Thus, the use of MC monocultures is a limitation to these experiments. Further investigations should include co-cultures, 3D cultures containing all three major glomerular cell subtypes, or isolated intact glomeruli studies to examine hRMC response to inflammatory stimuli. Additionally, these experiments used specific cytokines to stimulate MCs to examine cell responses. While using cytokines as the inflammatory stimuli was useful for identifying specific pathways and allowed for interrogating the role of GSL metabolism, the pathophysiological or *in vivo* relevance is uncertain. Further experimentation should incorporate more disease-specific stimuli, such as immune complexes and *in vivo* studies.

In conclusion, our results expand the mechanistic understanding of how GSL metabolism intersects with Ca^2+^ regulation, ER stress, and inflammation in MCs and suggest that GSL accumulation contributes to cytokine-induced ER stress (**Figure 6**). We speculate that cytokine stimulation (paracrine or autocrine) may elevate GSL metabolism through mechanisms that involve increased ceramide generation or activity of GSL synthesis enzymes, which can alter membrane lipid composition and downstream Ca^2+^ signaling. Future studies will be directed at delineating how cytokine-induced signaling pathways modulate GSL synthesis, the roles of specific GSL species (i.e. gangliosides), and whether these lipid alterations in turn influence ER Ca^2+^ or ER-mitochondrial dynamics, and modulation of UPR signaling in the context of renal inflammation. Importantly, targeting GSL biosynthesis may attenuate maladaptive organelle stress responses and protect against glomerular injury and be useful as a co-therapy in conjunction with anti-inflammatory therapeutics for a variety of renal inflammatory diseases, such as lupus nephritis.

## Data Availability

The NCBI Gene Expression Omnibus (GEO) accession number for the RNA-seq data are available at GSE311014. Custom R codes and data to support the analysis, visualizations, and functional enrichments are available https://github.com/BioinformaticsMUSC/NowlingLab_GLSproject/.
