# Supplementary material for: Cytokine-driven glycosphingolipid metabolism modulates endoplasmic reticulum calcium homeostasis in primary human renal mesangial cells
Source: Front Immunol. 2025 Dec 10;16:1688916. doi: 10.3389/fimmu.2025.1688916 (PMC12728008; doi:10.3389/fimmu.2025.1688916)
Supplement: Supplementary file 1 [file DataSheet1.pdf]

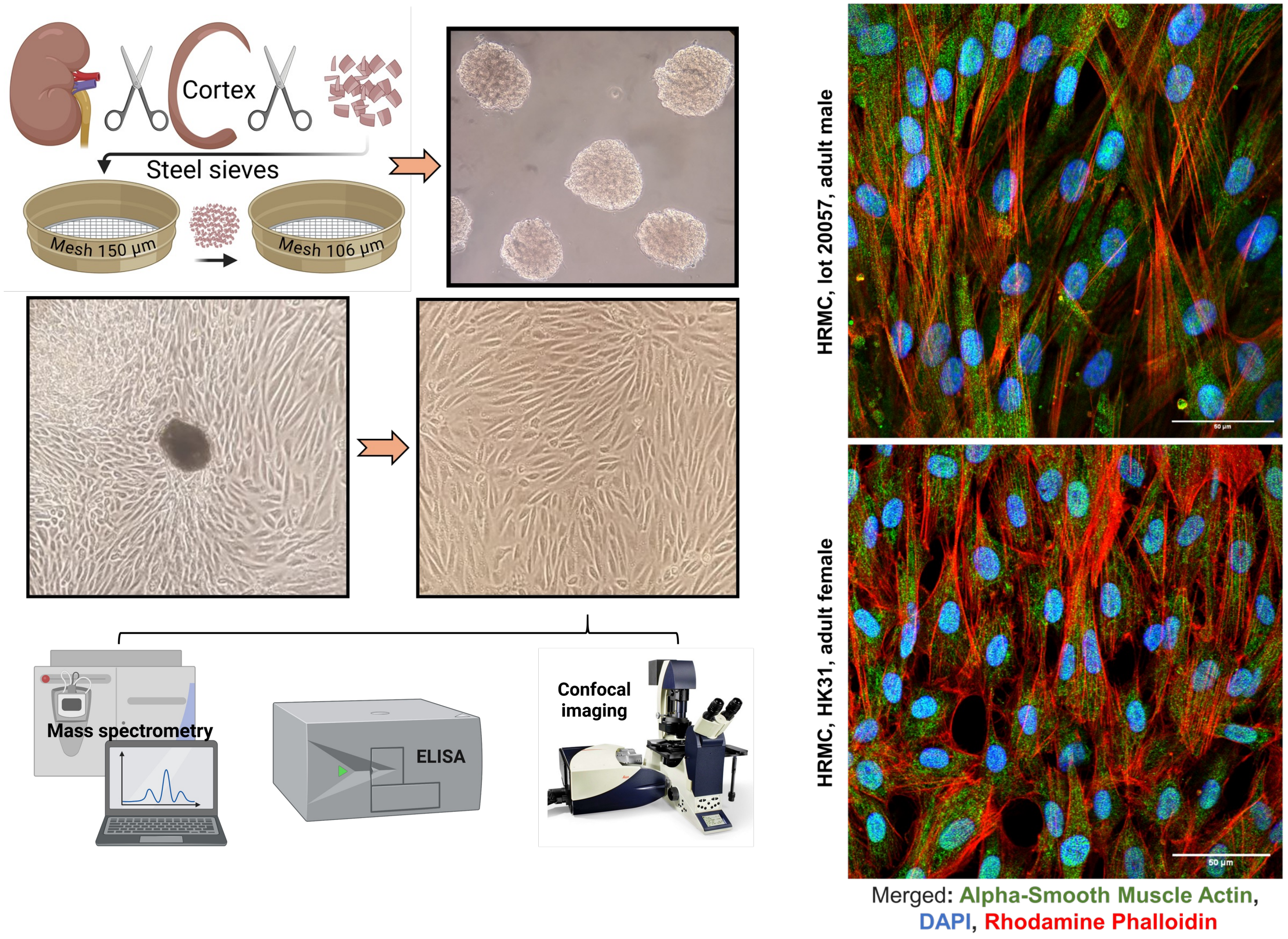

Fig. S1. Generation and characterization of hRMCs HK31 and HK32. Primary human renal mesangial cells (hRMCs) isolated from glomeruli in our lab were characterized visually for morphology and by staining with αSMA (green), rhodamine phalloidin (red), and DAPI (blue). Our hRMCs were compared to the commercially purchased hRMCs from ScienCell at passage 5 for both lines.

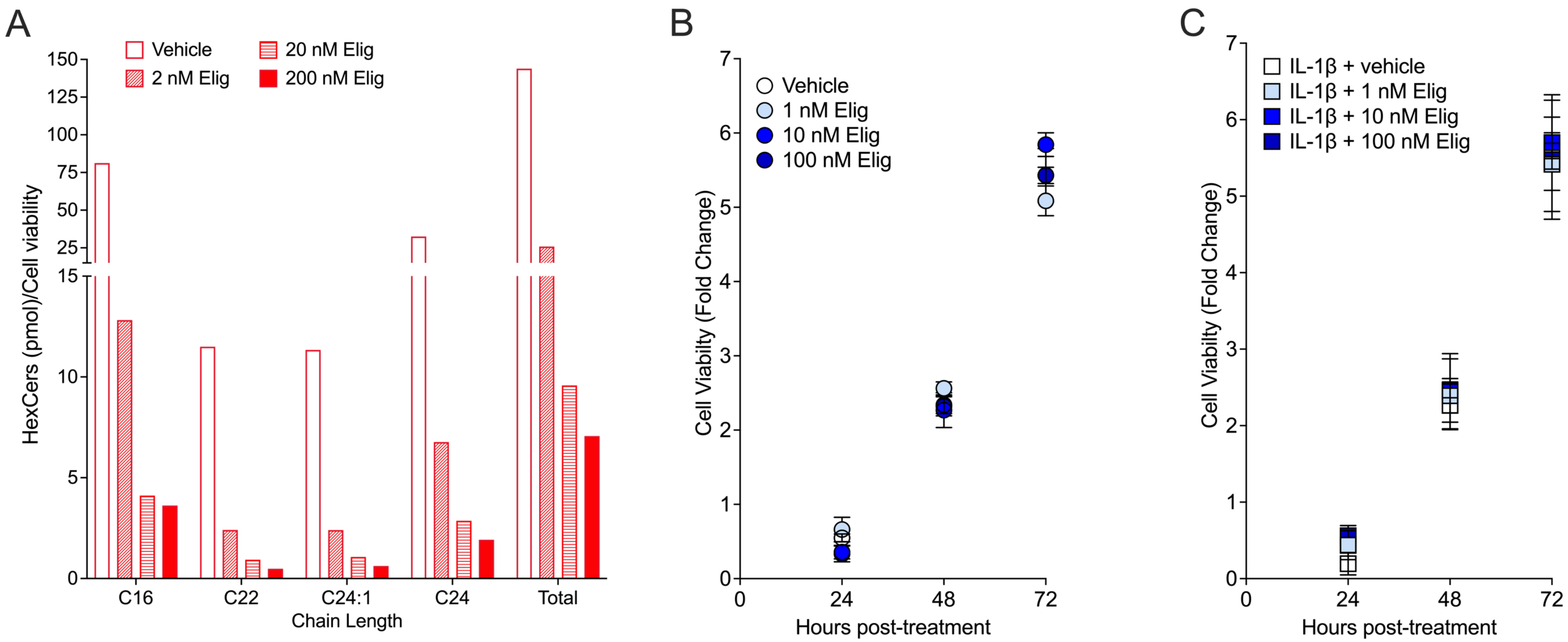

Fig. S2: Eliglustat dose-dependently reduces HexCers in hRMCs but does not impact proliferation/cell viability. A) hRMC lot 17544 were treated with vehicle or the concentration of eliglustat as indicated on the graph for 24h. HexCers were measured as in Fig. 2 and normalized to cell viability measured by alamar blue prior to cell collection. B) hRMCs lot 20018 were treated in quadruplicate with vehicle or the concentration of eliglustat indicated on the graphs. C) hRMCs lot 20018 were treated with vehicle or 25 ng/ml IL-1β in the absence pr presence of eliglustat. Cell viability using the alamar blue assay were measured at prior to treatment (0h) and at 24h, 48h, and 72h post-treatment, and presented as fold change compared to 0h. Data represent mean of quadruplicate wells +SD and are representative of results from other hRMC lots.

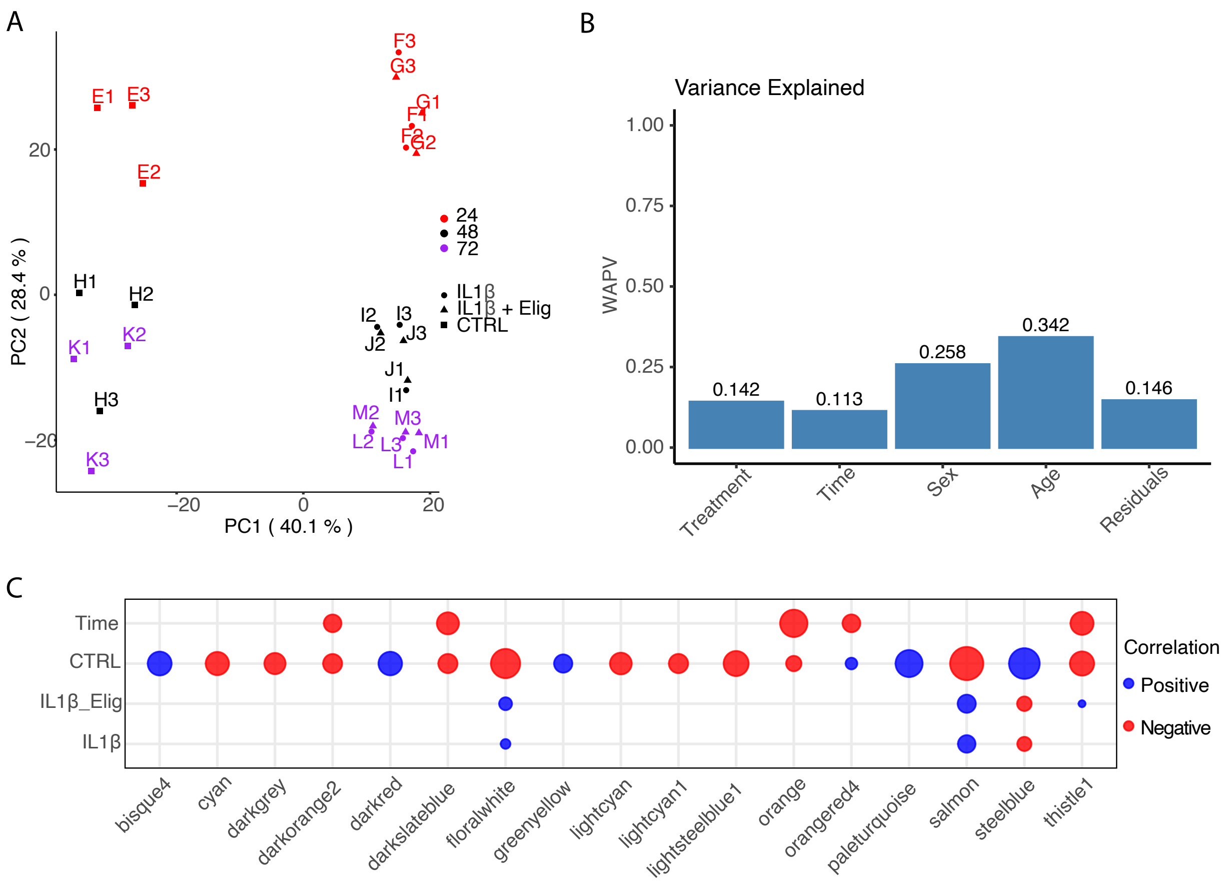

Fig. S3. Differentially expressed genes in response to treatment are similar across different hRMC lots. A) Principal component analysis of transcriptomes from IL-1β, IL-1β +eliglustat-treated, and untreated samples. Colors represent different time points, and shapes represent different treatments. B) Variance explained by each biological covariate included in the analysis. C) Bubble chart showing the significant correlations between the eigenvalues of co-expression modules and treatment/time. Colors indicate the direction of the correlation, and bubble size represents the strength of the correlation.
